# Mothers’ awareness towards child injuries and injury prevention at home: an intervention study

**DOI:** 10.1186/s13104-016-2031-5

**Published:** 2016-04-18

**Authors:** Anna Carlsson, Anna-Karin Dykes, Annkristin Jansson, Ann-Cathrine Bramhagen

**Affiliations:** Faculty of Health and Society, Department of Care Science, Malmö University, Jan Waldenströmsgata 25, 205 06 Malmö, Sweden; Faculty of Medicine, Department of Health Sciences Centre, Lund University, Lund, Sweden; Skane University Hospitals, Malmö, Sweden

**Keywords:** Awareness, Intervention studies, Primary prevention, Sense of Coherence

## Abstract

**Background:**

Most injuries to young children happen in the home. Therefore, this study aimed to investigate if extended individual information to mothers’ related to injury to children in the home and possible preventative actions has any effect on their awareness of the problem and if Sense of Coherence has an influence.

**Methods:**

This was a quasi-experimental designed intervention study with a comparison group. Extended individual information with empowerment as the approach was used.

**Results:**

Ninety-nine mothers of children under the age of 7 months participate. A questionnaire with sociodemographic data and questions regarding awareness towards prevention was used. Mothers who took part in the intervention significantly increased their awareness of the fact that child injuries take place at home when compared with the mothers in the comparison group, [OR 2.3, CI 1.3–4.3]. However, no significant improvement of awareness towards prevention was noted, neither any association to the mothers’ SOC-scores.

**Conclusion:**

This study showed that the intervention had a positive effect on mothers’ awareness towards the fact that child injuries are taking place at home, but it did not increase the mothers’ awareness towards prevention of child injury.

## Background

From an international perspective, incidents of child injuries and child mortality in Sweden are low [1]. According to Swedish Rescue Agency [2], several types of injury are the main cause of child mortality in Sweden among children between 1 and 14 years old and most injuries to young children (0–3 years old) take place in the home.

1 and 14 years old and most injuries to young children [0–3 years old] take place in the home. Most frequently it is boys of 1–2 years of age who suffer scald and burn injuries [3]. It is important to prevent burn and scald injury due to the pain of the treatment, and the risk for long-term rehabilitation [4, 5]. The injuries occurred mostly in the presence of an adult and were due to lack of supervision or the absence of injury preventative measures [6, 7]. Vladutin et al. [8] showed that mothers’ awareness towards child injury prevention was more strongly predictive among mothers with their first child rather than those with several children. In addition, research has stress the fact that one-third of all accidental injuries among children in the USA were preventable [9] and workshops in combination with home visits can improve the mother’s precautions against child injuries [10]. In Sweden, almost 100 % of all children aged 0–6 years participate in the Child Health Care [CHC] that focuses on the prevention of ill health. For all parents with children of 8 months, there is an additional focus on improving parent’s knowledge about precautions that need to be taken at home. This information is given on one occasion and includes information concerning the risks of drowning, scalding, burns, falls, and how to safely store chemicals, medicines and plastic bags. The rationale for this is the children’s growing physical mobility (e.g. their ability to crawl and walk) [11, 12].

Tengland [13] stressed that when professionals promote health care empowerment they should not have a controlling or paternalistic standpoint. Self-empowerment can, according to Laverack [14] and Adams [15], be achieved through education that gives the individual the tools for a healthier life-style. An unhealthy life style includes nutritional disorders (both malnutrition and over-nutrition), tobacco use, and harmful alcohol use, use of other harmful substances, high-risk sexual behavior, stress, common mental disorders, and all types of injury [16].

A human being’s inner strength is influenced by self-empowerment. Antonovsky [17, p. 19] defined a human being’s inner power by the concept ‘Sense of Coherence’ (SOC), which includes three main concepts: comprehensible, manageable and meaningful and how one sees one’s own world. A strong SOC indicates a better capacity to cope with difficult situations. Our hypothetical prediction was that a high SOC helps make a parent aware of the risks, and promotes them to take precautions against, possible child injuries.

Several reports have found that effective community-based interventional programmes potentially reduce the risk for a child being injured in their home, although few reviews gave any proof of effectiveness [11, 18]. Low socio-economic status has been found to be a risk factor for young children concerning the risk of being injured at home from scalding [19]. Furthermore, low parental education levels increased the risk for child injuries at home due to fewer precautions being taken by the parents [6]. In order to change the mothers’ awareness’s concerning accident prevention a focus on the individual family’s needs is necessary [20]. Therefore, the purpose of this study aimed to investigate if extended individual information has any effect on mothers’ awareness concerning child injury at home and preventive actions, and if Sense of Coherence has an influence.

## Methods

### Design and sample

This quasi-experimental designed intervention study with a comparison group took place in two different areas in a city in southern Sweden and focused on families with a low education level. Due to the possibility of a Hawthorn effect [21] in a randomized controlled trial from the same CHC, where the mothers in the control group might be influenced by the mothers in the intervention group, another design was chosen, namely a strategic comparison group from another similar CHC. In both areas only 18 % of the inhabitants had a higher education or university education. Also the occupational levels of the population in the city areas chosen showed similarities (38 respectively 39 % worked part or full time) and a majority of the inhabitants lived in rented apartments. The number of inhabitants not born in Sweden was 52 and 60 % respectively. The immigrant inhabitants where most often born in Iraq, Former Yugoslavia, Lebanon or Poland [22]. The two CHC centres with the largest number of registered children were chosen, one to be the intervention group (IG) and one to be the comparison group (CG). The CHC-nurses were informed about the aim of the study but not to which group their CHC was consigned.

The decision was made to create a sample of 100 mothers using a power calculation. The sample decreased to 99 since one mother answered the questionnaire twice. All mothers gave their written consent to participate. Fifty of the mothers were registered at one CHC (IG) and the other 49 mothers were registered at the other CHC (CG). The choice of which CHC was to be the IG respectively the CG was decided by lot. The mothers were identified through their children’s journals and were selected consecutively at the CHC by the first author. All the mothers with babies aged 4–7 months attending the CHC during a 6-month period were included and received a written invitation to participate in the study (n = 184). Three reminders were sent out. The CHC’s offered all the families standard information concerning prevention focusing on child injuries at home. This information aimed to prevent burns, fall injuries, poisonings and choking hazard.

### Measures

A pilot study with face validity, which included nine mothers, was performed in order to test the questionnaire. The questionnaire contained background questions such as, the mother’s awareness about child injuries and questions about awareness of child injury prevention at home. No changes in the questionnaire were necessary. The analysis of the current study did not involve the data from the pilot test.

Five questions concerned sociodemographic i.e. educational level, Swedish by origin, less than 4 years in Sweden, two children or more, and experience of historical injuries to children.

Furthermore, three statements focusing on child injury and the mothers’ awareness towards prevention at home (scores from 0–10/disagree-agree) were made (Table 1). The questionnaire was translated from Swedish into English, Arabic and Albanian.

**Table 1:** Baseline comparison between mothers’ awareness towards child injuries and injury prevention at home to mothers with/without experience of historical child injuries in the family (*n* = 99)

Variable (Missing = 4)	Baseline awareness	Injuries in children^a^	Without injuries in children^a^	*p* value*
Attitudes	Median^2^	(n) %	(n) %	
Many injuries to young children occur at home	5.00	(7) 35.0	(26) 34.2	NS
I have good and sufficient knowledge about suitable precautions to take to decrease the risk of child injuries at home	8.00	(9) 45.0	(38) 50.0	NS
I have taken sufficient precautions, to decrease the risk of child injuries at home	8.00	(7) 35.0	(34) 45.3	NS

^a^ Rates less than median

^2^ Scores from 0–10/disagree–agree

* Statistic significance at <0.05 (Mann–Whitney)

Moreover, Antonovsky's [17] instrument, Sense of Coherence (SOC-13) was used.

A systematic review [23] showed that this instrument is valid and reliable with a Cronbach’s alpha above 0.70 (0.70–0.92). Since the original version of SOC is in English it was translated into Albanian and Arabic (the most common used languages), and then back-translated by a second translator. The SOC instrument was already translated into Swedish.

### Intervention

The intervention consisted of two parts, a workshop and a home visit. The IG-mothers were invited to participate in a workshop which lasted between 30–60 min. The method is also presented in Carlsson et al. [10]. Two bilingual health communicators (Arabic and Albanian) assisted the first author in the workshop, which included 3–6 mothers in each workshop. Extended information, based on the individual mothers, with a focus on prevention of child injury at home, was performed during a home-visit and the first author collected data from the mothers about safety precautions taken at home. The mothers were also asked to mirror their awareness of the need for safety precautions. Two bilingual health communicators assisted when language problems arose. In order to increase the mothers’ awareness concerning injury prevention the pedagogic approach empowerment method was used, which means that the content of the discussions that took place during the intervention was determined by the mothers’ themselves. In order to inspire the mothers’ to reflect on prevention, the stated objective of the intervention was to find the mothers baseline knowledge level related to child injuries in the home and then to increase their knowledge on the subject. The mothers defined their problems and requirements, with support from the first author, in order to find solutions and ways of implementing suitable preventative actions. The home-visit in itself was a part of the intervention as in other studies [24, 25].

The follow-up home-visits took place when the children in both the IG and the CG had reached 9 months of age. Research assistants made on-site checks for any preventative safety measures that had been taken in the participant mother’s home. This included checking if the cooker was properly anchored, if there was heat protection around the cooker and oven, and further if there was a suitable high chair available to put the child in while the parent was working in the kitchen and that the electric kitchenware, (coffee machine, kettle, and iron) were kept out of reach for the children. The mothers then answered the baseline questionnaire once again.

### Statistics

The independent variable consisted of the extended individual information whereas the dependent variable was the mother’s awareness of child injury at home and preventions. The Mann–Whitney and χ^2^ method was used to assess dichotomized answers and for comparison between the groups, before and after the intervention. The Statistical Package for the Social Sciences programme (SPSS version 16.0) was used. A non-response analysis showed no significant differences in the background characteristics between the mothers in the IG and those in the CG. This study was approved by the Regional Ethics Board of Lund, Sweden (LU: 30/2008).

## Results

The participating mothers came from 25 different countries, most were from Iraq, Lebanon and former Yugoslavia. Background variables were collected and concordance was found between the mothers in both groups regarding (i) educational level, (ii) Swedish by origin, (iii) less than 4 years in Sweden, (iv) two children or more. A significant difference was noted between the groups regarding experience of historical injury to children. The children of the mothers in the intervention group had a significantly higher number of historical injuries (n = 17) than those in the comparison group (n = 3), (*p* < 0.001).

### Children who had been injured historically

Children in both groups, who had a history of injuries in the home, 50 % (n = 10), received their injuries from scald and burn accidents, whereas 30 % (n = 6) had fallen from furniture, and 20 % (n = 4) had suffered from eye or head injury, or medicine poisoning.

No significant difference was found in a comparison between mothers’ awareness towards child injuries and injury prevention in the home at baseline, or regarding mothers with or without experience of historical child injuries in the family (Table 1).

When comparing the effect of SOC-scores assessed at baseline concerning awareness, no influence was found in a comparison between groups at follow-ups. The association of a high/low SOC-score with the mothers’ awareness towards child injuries and injury prevention at home was not proven in the baseline-assessed awareness or in the comparison between the IG and the CG of mothers at follow-ups (Table 2).

**Table 2:** Baseline comparison between mothers’ awareness towards child injuries and injury prevention at home and mothers with high/low SOC-score, presented with statistical significance between the intervention group (IG) and the comparison group (CG) of mothers (n = 99)

Variable	Baseline awareness median	SOC ≥ 65^c^	SOC ≤ 64^c^	IG compared to CG
Awareness		(n) %^a^	(n) %^a^	p value*
Many injuries to young children occur at home^b^	5.00	(20) 44.4	(25) 55.6	NS
I have good and sufficient knowledge about suitable precautions to take to decrease the risk of child injuries at home^b^	8.00	(20) 54.1	(17) 45.9	NS
I have taken sufficient precautions to decrease the risk of child injuries at home^b^	8.00	(20) 51.3	(19) 48.7	NS

^a^ Rates less than median

^b^ Scores from 0–10/disagree-agree

^c^ Total score of SOC-13 (possible score: 13–91)

* Statistic significance at <0.05 (χ²)

However, concerning the subject; ‘many injuries to young children occur at home’ the awareness in the IG had improved significantly after intervention compared to baseline (*p* < 0.025). A comparison between those who took part in the IG and those of the CG indicated that the IG mothers had increased their awareness to a greater extent [OR 2.3, CI 1.3–4.3]. The IG was statistically used as the reference group [OR 1.0], (Table 3).

**Table 3:** Mothers’ awareness towards child injuries at home and injury prevention before and after individual-based information, between the Intervention group (IG) and the comparison group (CG), (*n* = 99)

Statement	Intervention group n = 50			Comparison group n = 49			IG compared to CG
	Baseline awareness (n) %	Follow-up (n) %	*p* value	Baseline awareness (n) %	Follow-up (n) %	p value*	OR (95 % CI)
*Missings*	*(0) 0*	*(14) 28*		*(3) 6*	*(17) 34*		
Many injuries to young children occur at home^b^	Median 5.00	Median 7.00	0.025*	Median 5.00	Median 5.00	0.255	2.3 (1.3–4.3)^a^
I have good and sufficient knowledge about suitable precautions to take to decrease the risk of child injuries at home^b^	Median 8.00	Median 8.00	NS	Median 8.00	Median 9.00	NS	
I have taken sufficient precautions, to decrease the risk of child injuries at home^b^	Median 8.00	Median 9.00	NS	Median 8.00	Median 9.00	NS	

^a^ IG = reference group (OR = 1.0)

^b^ Scores from 0–10/disagree-agree

* Statistic significance at < 0.05 (Mann–Whitney)

## Discussion

This study showed that an intervention with extended individual information and home visits concerning the prevention of child injury at home had a significant positive effect on mothers’ awareness towards child injury. Since research has shown that approximately 30 % of all unintentional injuries among children can be prevented [9] studies focusing on this issue must be considered important. In Sweden, the most vulnerable group is boys between 1–2 years of age who are at risk for being exposed to scald and burn injuries [3] and in order to highlight prevention of these injuries this study focuses on children of below 1 year.

A review study has shown that parental intervention seems to be effective [18] and there is good evidence that home visiting might have an impact on decreasing the number of injuries among children [26]. Babul et al. [24] showed that parental safety behavior increased after the intervention however, no significant reduction of child injury was found in the parent’s reports. In order to change the mothers’ awareness of prevention an essential factor is, to make the mothers’ more aware of the problem. Our study showed that the mother’s awareness regarding child injury had increased, even though we did not succeed in improving their prevention activities actions. Even so, this intervention must be considered important and should be developed further.

After the study questionnaire was developed, a pilot test with face validity was performed and any obstacles preventing a full understanding of the questions due to language were decreased after the questionnaire was presented in English, Albanian and Arabic.

In all, 29 % of the mothers were missing in the follow-up but in a comparison of the mothers who completed the study and the non-responders, little variation and no significant difference was found. This study showed that the mothers’ awareness towards child injuries and injury prevention was not influenced by their SOC-scores but there is a likelihood that the sample size in the current study was too small to enable associations to be made.

The baseline characteristics of the mothers in the IG and the CG respectively were similar except for the historical injuries that had happened to a greater number of the children in the IG. This might have been a reason why the IG, after the intervention, had an increased awareness concerning the fact that many young children are injured at home. Yet the intervention showed no significant effect on awareness of prevention among the IG. This is in line with the results from Vladutin et al. [8], which showed that parental experiences reduce the impact of their awareness as their behaviour becomes influenced by their experiences and expected effects.

Furthermore, there might also be a limitation of the study by the fact that mothers with a history of experiences concerning injury among their children could have been more inclined to have increased awareness, compared to mothers lacking such experiences. The high median scores reached in questions concerning mothers’ sufficient knowledge and the taking of sufficient precautions was found in both the IG and the CG. It might be explained as an effect of the mothers’ reading the questionnaire and therefore reflecting on child accident prevention. Reflection upon awareness toward the prevention of child injury at home could possibly have influenced the mothers’ to become more aware. Furthermore, the reflection might be more pertinent in relation to the mothers’ awareness of the possible risk for injury at home. Perhaps the answer alternatives ‘sufficient’ and ‘good’ were not optimal and hence misled the mothers. Another explanation for why the two questions above did not show significant improvement of the mothers’ awareness might be that they were already high at baseline. Some mothers also stated that their knowledge had been insufficient before receiving the extended individual information. One explanation for this may be that after the intervention mothers became aware of what they did not know prior to the intervention.

Furthermore, the workshop might have increased the mother’s understanding about their own knowledge, which led to their awareness that they overvalued their knowledge at baseline.

The intervention consisted of home-visits offering empowerment and extended individual information based on the individual person attending. To overcome some of the risk for bias, the first author made all the interventions.

This study cannot verify which part of the intervention it was that increased the mothers’ awareness of the fact that child injuries occur at home. WHO and UNICEF recommend home visits in the baby’s first week of life [27]. There are a number of studies and research reviews published on the effect of how home visits can reduce the risk of accidental injuries in the home and how home visits may encourage parents to reduce potential hazards in the home [26, 28]. Although arguments have been made for the CHC to increase the number of home-visits there has been a trend towards the reduction of home-visits over the last years which could a lack of time. This study showed that the intervention had a positive effect on mothers’ awareness towards the fact that child injuries are taking place at home, but it did not increase the mothers’ awareness towards prevention of child injury. However, an essential fact in order to improve mothers’ awareness of prevention of child injuries at home is, to make them fully aware of the problem. This is an important task in the work of child health care aimed to prevent child injuries.

## Conclusion

This study showed that the intervention had a positive effect on mothers’ awareness towards the fact that child injuries are taking place at home, but it did not increase the mothers’ awareness towards prevention of child injury. The clinical implications are that Child Health Care Services has a huge challenge in developing interventions in order to prevent child injuries and to make the mothers’ aware of the importance of prevention.
